# 1,1′-Biisoquinolines—Neglected Ligands in the Heterocyclic Diimine Family That Provoke Stereochemical Reflections

**DOI:** 10.3390/molecules26061584

**Published:** 2021-03-13

**Authors:** Edwin C. Constable, Richard M. Hartshorn, Catherine E. Housecroft

**Affiliations:** 1 Department of Chemistry, University of Basel, BPR 1096, Mattenstrasse 24a, CH-4058 Basel, Switzerland; catherine.housecroft@unibas.ch; 2 School of Physical and Chemical Sciences, University of Canterbury, CT1 1PL Christchurch, New Zealand; richard.hartshorn@canterbury.ac.nz

**Keywords:** Ligand, 1,1′-biisoquinoline, bidentate, atropisomerism, chirality, stereochemical nomenclature

## Abstract

1,1′-Biisoquinolines are a class of bidentate nitrogen donor ligands in the heterocyclic diimine family. This review briefly discusses their properties and the key synthetic pathways available and then concentrates upon their coordination behaviour. The ligands are of interest as they exhibit the phenomenon of atropisomerism (hindered rotation about the C1–C1′ bond). A notation for depicting the stereochemistry in coordination compounds containing multiple stereogenic centers is developed. The consequences of the chirality within the ligand on the coordination behaviour is discussed in detail.

## 1. Introduction

**Figure 1:**
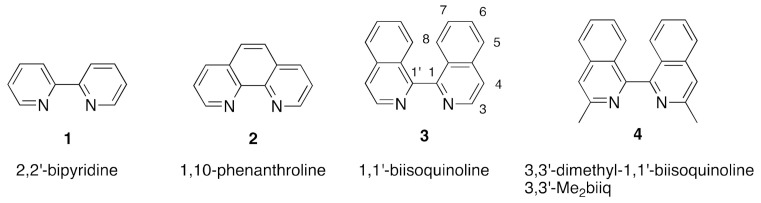
The structures of the „parent” heterocyclic diimines 2,2′-bipyridine (**1**) and 1,10-phenanthroline (**2**) together with that of 1,1′-biisoquinoline (**3**), the subject of this review. The IUPAC numbering scheme for derivatives of 1,1′-biisoquinoline is indicated and also shown is an example of the abbreviated nomenclature adopted in this review using 3,3′-Me_2_biiq as an example of an abbreviation for 3,3′-dimethyl-1,1′-biisoquinoline (**4**).

The heterocyclic diimines 2,2′-bipyridine (**1**) and 1,10-phenanthroline (**2**) and their derivatives are among the commonest chelating nitrogen donor ligands in coordination and organometallic chemistry (Figure 1) [1,2,3,4,5,6,7,8,9,10]. The heterocyclic diimine metal-binding domain is widely used as a scaffold in supramolecular chemistry and in interfacial science. Compounds **1** and **2** are members of a much larger series of bis(heterocycles) incorporating the N=C–C=N chelating metal-binding motif. This review is concerned with the coordination behavior of one of the less well-known members of this series, 1,1′-biisoquinoline (**3**) (Figure 1). The 1,1′-biisoquinolines are of especial interest as steric interactions between nitrogen lone pairs, substituents on the nitrogen and between H8 and H8′ favour a non-planar geometry in both the free ligands and their chelated metal complexes. The consequences of this are discussed in Section 2.

Derivatives of **3** will generally be named in an abbreviated form utilizing the short-form biiq with the substituents located using the numbering scheme indicated in Figure 1; for example, 3,3′-dimethyl-1,1′-biisoquinoline (**4**) would be written 3,3′-Me_2_biiq (Figure 1). We note that various abbreviations have been used for 1,1′-biisoquinoline in the literature, including biq and bq, which are also used by other authors for 2,2′-biquinoline.

This review concentrates upon the properties of this interesting class of compounds as ligands and also includes a non-comprehensive overview of their properties and major synthetic routes for their preparation. Condensed and reduced derivatives of biiq are excluded from this review. Much of the interest in 1,1′-biisoquinolines relates to their stereochemical and stereogenic properties, in particular they belong to the class of diaryls which exhibit atropisomerism. In the course of preparing this review, we became aware of a need for a clear notation to describe the stereochemical configuration in coordination compounds containing multiple stereogenic features (centers or axes) and/or chiral ligands. All structural information and figures have been extracted from the Cambridge Structural Database [11] using the Conquest search [12] and Mercury visualization software [13].

## 2. Atropisomerism

### 2.1. Atropisomerism 

Before considering the chemistry of the 1,1′-biisoquinolines themselves, it is worth reviewing the concept of atropisomerism. 

**Figure 2:**
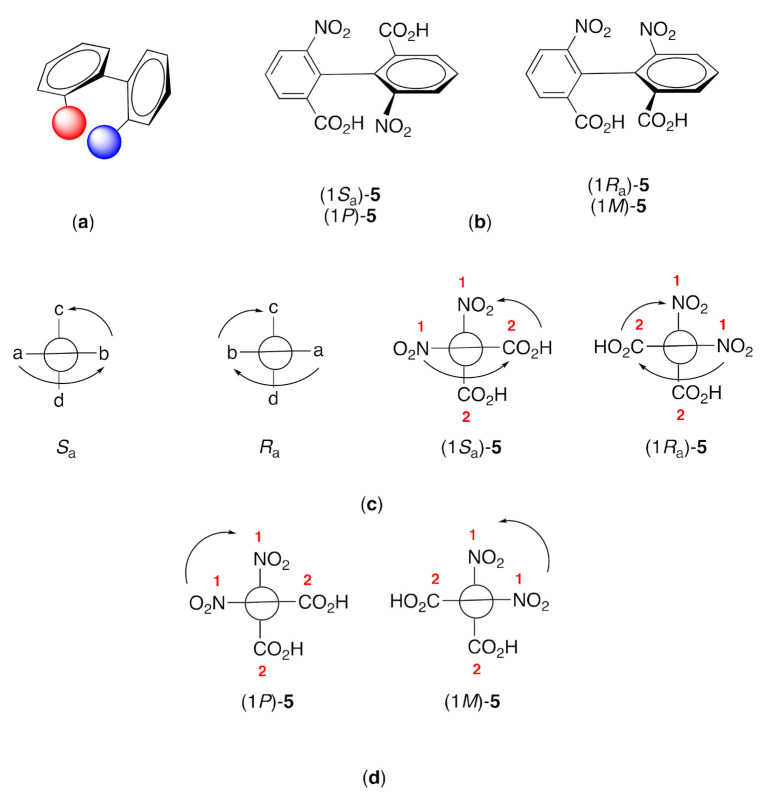
(**a**) Steric interactions between the red and blue substituents in biaryls can lead to atropisomerism with the chiral axis being the interannular C–C bond; (**b**) the first atropisomeric compounds to be resolved were the enantiomers of 6,6′-dinitro-[1,1′-biphenyl]-2,2′-dicarboxylic acid; (**c**) the Cahn-Ingold-Prelog scheme can be extended to the nomenclature of axially chiral compounds using the usual priority rules for an „extended tetrahedron”; (**d**) but IUPAC recommends the use of *P* and *M* assigned using Cahn-Ingold-Prelog priorities as described in the text.

Atropisomers are stereoisomers which arise as a result of hindered rotation about a single bond, and atropisomerism is a form of axial chirality [14]. In the classical period of organic chemistry, it was assumed that rotation around C–C bonds was, to all intents and purposes, without an energy barrier. Atropisomerism is commonly observed in compounds in which aromatic rings are connected by a single C–C bond, with the barrier to rotation arising from interactions between substituents on the aromatic rings (Figure 2a). The first compounds exhibiting atropisomerism to be resolved were 6,6′-dinitro-[1,1′-biphenyl]-2,2′-dicarboxylic acid (Figure 2b) and 4,4′,6,6′-tetranitro-[1,1′-biphenyl]-2,2′-dicarboxylic acid [15], although the possibility of such forms of enantiomerism has been predicted earlier by a number of authors [16,17,18]. It was left to Werner Kuhn to introduce the term atropisomerism (*atropisomerie*), derived from the Greek *ατροπος* (atropos) meaning “without turn”, to describe this phenomenon [19].

IUPAC recommends the use of the stereochemical descriptors *P* and *M* rather than the more commonly encountered *R*_a_ and *S*_a_ to denote the stereogenic axis in the Preferred IUPAC Name (PIN) of axially chiral compounds (Rules P-92.1.2.1.2, P-92.1.2.2, P-92.1.2.2.1 and P-93.5. 7.1 and Figure 2b) [20]. Standard Cahn-Ingold-Prelog rules are used to determine the priority of substituents treating the compounds as „extended tetrahedra” with *R*_a_ and *S*_a_ defining a clockwise or anticlockwise sequence (Figure 2c) [21,22,23,24]. In particular, those rules are used to identify the order of the two groups at one end (a and b in Figure 2c), and the higher ranked group (c) of the two at the other end. 

The descriptors *P* and *M* refer to *p*lus and *m*inus, relating to a right- or left-handed helicity, respectively. The substituents a, b, c and d are arranged in pairs looking along the chirality axis. The highest priority substituent in each pair is identified, and the chirality is described as *M* if the path between these two is anticlockwise, and as *P* if it is clockwise (Figure 2d). We believe that this is easier to apply than the *R*_a_/*S*_a_ approach.

**Figure 3:**
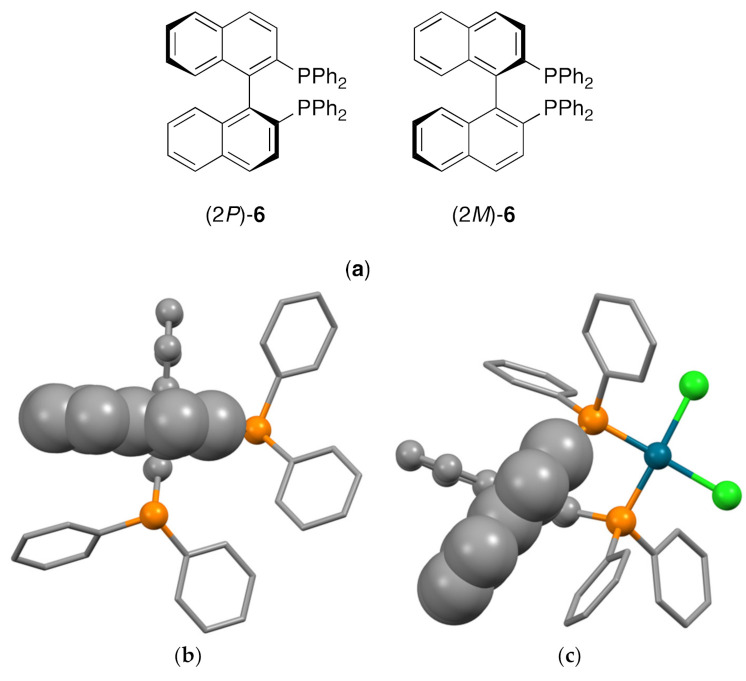
(**a**) The two atropisomeric forms of BINAP, [1,1′-binaphthalene]-2,2′-diylbis(diphenylphosphane), **6**; (**b**) in the free ligand the naphthalene rings are near-orthogonal, minimizing mutual interactions between the substituents, hydrogen atoms omitted for clarity, phosphorus shown as balls, the naphthalene closest to the viewer in 70% space-filling and the remote naphthalene in ball and stick representation; (**c**) in the complex [Pd{(2*P*)-**6**}Cl_2_] the interannular angle between the naphthalene rings is reduced to 69.9^o^ allowing Pd–P bond lengths of 2.244 Å and a P–Pd–P bite angle of 92.68°, hydrogen atoms omitted for clarity, phosphorus, palladium and chlorine shown as balls, the naphthalene closest to the viewer in 70% space-filling and the remote naphthalene in ball and stick representation.

In practical terms, for the isolation of enantiopure forms, the interconversion of atropisomers should exhibit a half-life of at least 1000 s at 300 K which corresponds to an energy barrier for rotation of 93 kJ mol^−1^ [25,26]. Inorganic chemists are probably most familiar with the phenomenon of atropisomerism in the context of chelating bisphosphane ligands such as BINAP ([1,1′-binaphthalene]-2,2′-diylbis(diphenylphosphane), **6**, Figure 3a) [27,28,29,30,31,32,33,34,35]. It is instructive to use ligand **6** to illustrate the consequences of coordination. The free ligand is non-planar as a consequence of the interactions between the 2-PPh_2_ and 2′-PPh_2_ substituents and also between H8 and H8′; this is seen in the solid-state structures of (2*P*)-**6** (*P*2_1_) [36,37] (2*M*)-**6** (*P*2_1_) [37,38] and *rac*-**6** (*C*2/*c*) [37,39] which have all been reported. In all cases the two aromatic rings of the 1,1′-binaphthalene scaffold are close to orthogonal in the solid state with torsion angles C2–C1–C1′–C2′ in the range 91.536–93.429°. Upon coordination, the conformation of the ligand will be influenced not only by the steric interactions within the ligand, but also by the optimization of the metal-donor atom bond lengths and ∠P–M–P bond angles. This is clearly seen in the complex [Pd{(2*P*)-**6**}Cl_2_] (Figure 3c), in which the torsion angle C2–C1–C1′–C2′ is reduced to 69.9° enabling optimal Pd–P bond lengths of 2.244 Å, a ∠P–Pd–P bite angle of 92.68° and a concomitant reduction in the H8–H8′ distance of ~0.2Å [40]. 

### 2.2. Atropisomerism in 1,1′-Biisoquinolines 

**Figure 4:**
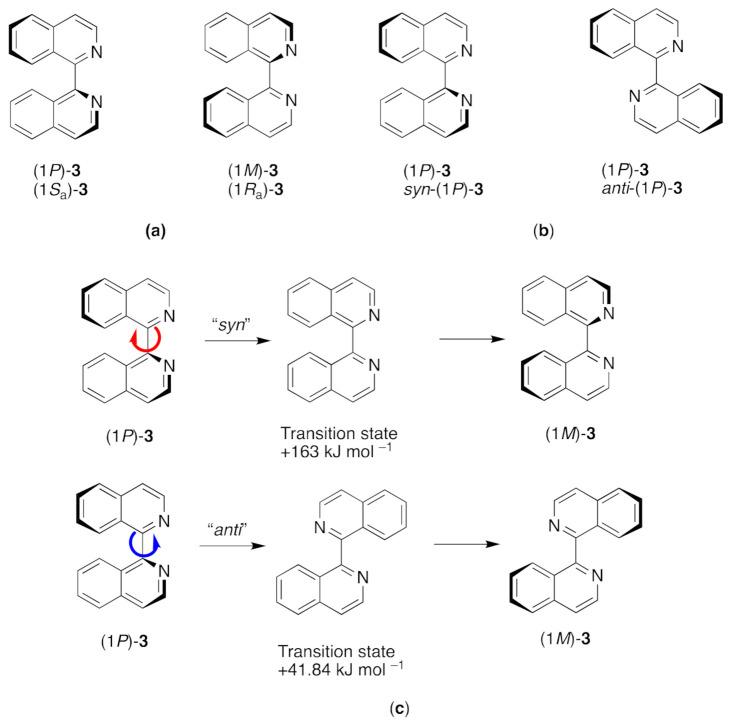
(**a**) The two enantiomeric (atropisomeric) forms of 1,1′-biisoquinoline; (**b**) each enantiomer can adopt a *syn* or an *anti* conformation (*syn* N–C–C–N torsion angle < 90°, *anti* N–C–C–N torsion angle > 90°); (**c**) racemization can involve a rotation through a *syn* (the red arrow denotes the direction of rotation of the top quinoline) or an *anti* (the blue arrow denotes the direction of rotation of the top quinoline) conformation. The latter is favored as the H8 and H8′ atoms do not have to pass through each other.

1,1′-Biisoquinolines have a chiral axis defined by the C1–C1′ bond (Figure 4a). Attempts to resolve the parent compound **3** via the tartrate salt of the protonated ligand were unsuccessful, with mutorotation occurring in aqueous solution within 80 s in 0.3 M hydrochloric acid [41]. Chiral stationary phases [42] and complexation with chiral palladium complexes [43,44] have been used for the resolution of **3** and 8,8′-dialkyl derivatives [45,46]. Steric interactions are increased with the presence of substituents on the nitrogen atoms, and, in contrast to **3** itself, 1,1′-biisoquinoline *N,N’*-dioxide [47,48] and 8,8′-(MeO)_2_biiq *N,N’*-dioxide [42] can also be resolved on chiral solid phases. Somewhat unexpectedly, the rate of racemization increases in the sequence 8,8′-Me_2_biiq < 8,8′-Et_2_biiq < 8,8′-*^i^*Pr_2_biiq, with activation energies at 303 K being close to 100 kJ mol^–1^ [43,46]. Various chiroptical correlations have been used to show that the (+)-forms of 1,1′-biisoquinolines possess the (1*M*) absolute configuration (Figure 4) [42,45,46,49,50,51].

Computational chemical studies at the MOMM (molecular orbital molecular mechanics) level have been reported for the free ligand biiq and confirm that the *anti* pathway for racemization is favoured over the *syn* pathway, with the barrier for *syn* rotation being estimated at 163 kJ mol^–1^ and that for *anti* rotation as 41.84 kJ mol^–1^ [52].

**Figure 5:**
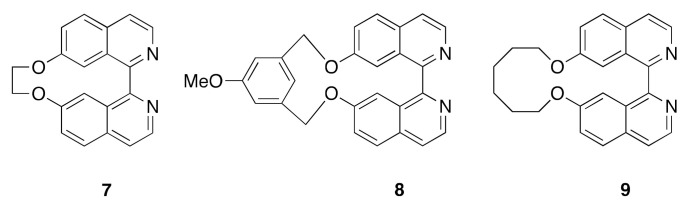
Macrocyclic ligands incorporating 1,1′-biisoquinoline metal-binding domains.

Macrocyclic ligands (Figure 5) incorporating 1,1′-biisoquinoline subunits have been of interest in studying the influence of the additional ring constraints on racemization. Compounds **7** and **8** were prepared from the appropriate open-chain bis(1-haloisoquinolines) using an Ullmann reaction and resolved using HPLC on a chiral phase [53]. Compound **7** racemized in boiling EtOH with a *t*_1/2_ of 64 min (ΔG^‡^ 110 kJ mol^–1^) whilst **8** is configurationally stable. The larger ring macrocycle **9** exhibits rather more interesting behaviour and displays a dynamic kinetic resolution on treatment with a chiral acid. Rapid racemization takes place in polar and protic solvents, as well as under acidic conditions [54].

## 3. Synthesis of 1,1′-Biisoquinolines

**Scheme 1:**
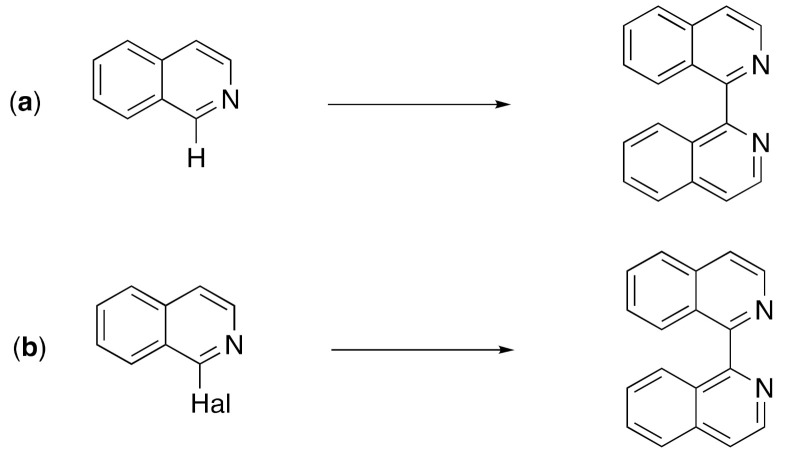
The commonest methods for the synthesis of 1,1′-biisoquinolines are (**a**) the oxidative dimerization of isoquinolines, and (**b**) the coupling of 1-haloisoquinolines. The various methods are described in detail in the text.

The standard works on the synthesis and properties of quinolines and isoquinolines do not contain significant details of the 1,1′-biisoquinolines [55,56,57,58]. Although a range of „exotic” methods of synthesis has been reported [55,59,60,61,62,63,64,65,66,67,68,69], the commonest methods relevant to the bulk preparation of ligands for use in coordination chemistry are the oxidative dimerization of isoquinolines (Scheme 1a) and the coupling of 1-haloisoquinolines (Scheme 1b) [70,71]. 

The oxidative dimerization of isoquinoline with LDA/HMPA (LDA = Li(N*^i^*Pr_2_), HMPA = hexamethylphosphoramide) in diethyl ether is reported to give **3** in 35–55% yield [72,73,74], better than the 26.3% yield obtained from the reaction with sodium naphthalenide [75,76] but not approaching the 87% obtained from the reaction with MgCl(TMP) (HTMP = 2,2,6,6-tetramethylpiperidine) [74]. The compound is also obtained from the treatment of isoquinoline with LDA/TMEDA (TMEDA = *N*^1^,*N*^1^,*N*^2^,*N*^2^-tetramethylethane-1,2-diamine) in diethyl ether, although no yield was reported [77]. The dehydrogenation reactions of isoquinoline with transition metals are not especially effective: reaction with Pd–C [78] or Ni–Al_2_O_3_ [79] do not yield **3** and only trace amounts are obtained with Raney nickel [80]. The best yields (<10%) are obtained with Rh-C catalysts [80]. 

The first reported synthesis of **3** was from a classical Ullmann reaction of 1-bromo-isoquinoline with copper metal at 210–230 °C [81]. The reaction of 1-bromoisoquinoline with either sodium or magnesium does not yield **3** [79]. The coupling of 1-haloisoquinolines has been achieved with Zn–[NiBr_2_(PPh_3_)_2_] [47,82], Zn–[NiBr_2_(PPh_3_)_2_]–(Et_4_N)I [70,83], Zn–NiCl_2_–PPh_3_ [43,44,45,46,84,85,86,87] or In–[Pd(PPh_3_)_4_] [88] catalysts. Substituted derivatives of biiq which have been prepared using this approach include 3,3′-Me_2_biiq [84], 8,8′-Me_2_biiq [45,46], 3,3′-Et_2_biiq [84], 8,8′-Et_2_biiq [45,46], 3,3′-(*^i^*Pr)_2_biiq [84], 8,8′-(*^i^*Pr)_2_biiq [45,46], 8,8′-(*^t^*Bu)_2_biiq [45,46], 3,3′-Ph_2_biiq [84], 3,3′-(2-py)_2_biiq [84], 6,6′-(MeO)_2_biiq [84], 3,3′-(MeO_2_C)_2_biiq [83], 3,3′-(HO_2_C)_2_biiq [83], 4,4′-(C*_n_*H_2*n*+1_O)_2_biiq (*n* = 1–11, 13, 17) [86], 4,4′-{*^n^*ROC_6_H_4_O(CH_2_)_n_O}_2_biiq (*R* = C_6_H_13_, C_8_H_17_; *n* = 4, 6) [86]. An interesting modern variant on the Ullmann reaction utilizes Au-Pd nanochain networks; **3** is obtained in 77% yield from 1-chloroisoquinoline on treatment with the Au-Pd nanochain networks and K_2_CO_3_ in aqueous ethanol [89]. Other noteworthy variants are seen in the reaction of 1-iodoisoquinoline with *^t^*BuLi and MnCl_2_ followed by aerial oxidation, which gives **3** in 78% yield [90] or the preparation of **3** from 1-bromoisoquinoline in 30% yield by reaction with *^n^*Bu_6_Sn_2_ and [Pd(PPh_3_)_4_] [91].

**Scheme 2:**
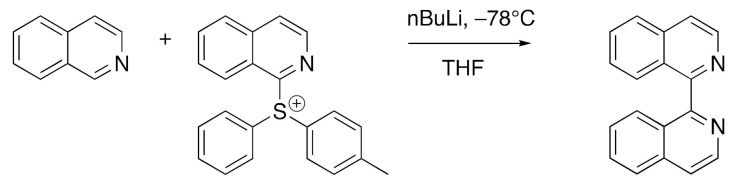
A new synthetic method with potential for the preparation of asymmetrically substituted 1,1′-biisoquinolines [92,93].

One method likely to be of future application in the synthesis of asymmetric 1,1′-biisoquinolines is presented in Scheme 2: the parent compound **3** is obtained in 46% yield in gram-scale preparations [92,93]. 

The treatment of isoquinoline *N*-oxides with K(O*^t^*Bu) and AIBN (AIBN = azobisisobutyronitrile) in DMF or THF has been used for the preparation of **3**, 3,3′-Me_2_biiq, 5,5′-Cl_2_biiq and 6,6′-Br_2_biiq in 54–77% yield [94]. Similarly, the reaction of isoquinoline *N*-oxides with Li(O*^t^*Bu) in chlorobenzene at 120 °C gives **3** in 50% yield [95]. Both strategies are potentially of interest for future research in the field.

### 3.1. 1,1′-Biisoquinoline N,N’-Dioxides 

**Figure 6:**
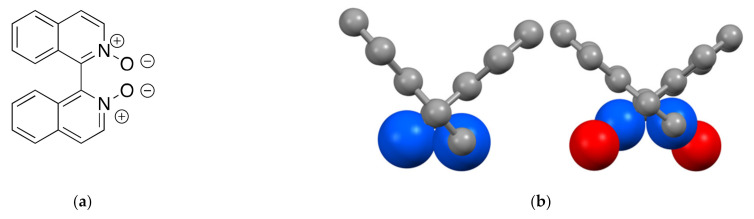
(**a**) 1,1′-Biisoquinoline *N,N’*-dioxide; (**b**) in the solid state, the torsion angle N1–C1–C1′–N2′ increases from 90.96° in (1*P*)-1,1′-biisoquinoline [108] to 101.64° (1*P*)-1,1′-biisoquinoline *N,N’*-dioxide [98] showing the importance of oxygen–oxygen interactions. The nitrogen and oxygen atoms are depicted with 70% space-filling size to emphasize the stereochemistry.

1,1′-Biisoquinoline *N,N’*-dioxides (Figure 6a) are readily prepared from the parent 1,1′-biisoquinolines using classical methods of *N*-oxidation involving peracetic acid or MCPBA (MCPBA = 3-chloroperbenzoic acid) [47,48,96] and have been resolved using chiral HPLC. The compounds appear to be configurationally stable. Resolved 1,1′-biisoquinoline *N,N’*-dioxides have found some applications as enantioselective organic catalysts [49,50,97,98,99,100,101,102,103,104,105,106,107]. The complex (1,1′-biisoquinoline *N,N’*-dioxide)dichloridopalladium is an effective catalyst for Suzuki cross-couplings and hydroxyarylation reactions [96].

The conversion of 1,1′-biisoquinoline to its *N*,*N*’-dioxide is expected to increase the steric interactions between the aryl subunits as the oxygen–oxygen interactions are likely to have a greater steric demand than the lone pair–lone pair interaction in the parent compound. Only a single example of a 1,1′-biisoquinoline *N,N’*-dioxide has been structurally characterized in a co-crystal of (1*P*)-1,1′-biisoquinoline *N,N’*-dioxide and (1*M*)-[1,1′-binaphthalene]-2,2′-diol [98]. A comparison of the N2–C1–C1′–N2′ torsion angle in 1,1′-biisoquinoline (90.96°) [108] to that in 1,1′-biisoquinoline *N,N’*-dioxide (101.64°) [98] (101.64°) shows that the oxygen–oxygen interactions dominate and these substituents adopt an *anti* comformation (Figure 6b).

### 3.2. Quaternized 1,1′-Biisoquinolinum Salts

**Figure 7:**
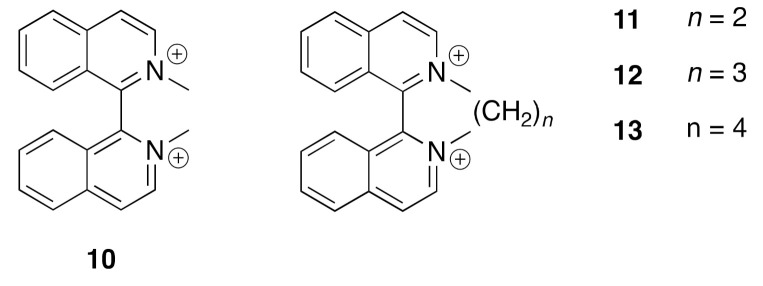
Some 1,1′-biisoquinolinium cations.

A number of quaternized 1,1′-biisoquinolinium salts have been prepared and characterized using classical alkylating agents (Figure 7) [109,110,111]. Compounds (**10**)(I)_2_, (**11**)(Br)_2_, (**12**)(Br)_2_ and (**13**)(Br)_2_ exhibit a blue chemiluminescence in basic solution that is enhanced in the presence of H_2_O_2_. The reaction products are *N,N’*-linked isoquinolin-1(2*H*)-ones arising from cleavage of the C1-C1′ bond [111,112,113]. Several of these compounds have been structurally characterized and these are presented in Section 4.1.

## 4. Some Relevant Physical and Structural Properties of 1,1′-Biisoquinolines

### 4.1. Structural Studies 

**Figure 8:**
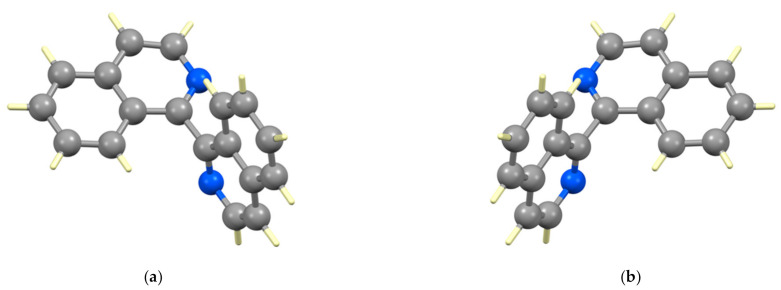
Both enantiomers of 1,1′-biisoquinoline are observed in the crystal structure of *rac*-biiq; (**a**) (1*M*)-biiq and (**b**) (1*P*)-biiq [108].

A number of 1,1′-biisoquinolines and derivatives has been structurally characterized and relevant data are presented in Table 1 and Table 2. Table 1 presents „simple” 1,1′-biisoquinolines and Table 2 comprises quaternized and related derivatives. Structurally characterized metal complexes are discussed individually in the relevant sections. In all cases, the individual biisoquinoline ring systems are near-planar and torsion angle N2–C1–C1′–N2′ is used as a measure of the relative orientation of the rings about the interannular C–C bond that defines the chiral axis. It is also worth commenting that no structure of an enantiopure 1,1′-biisoqiuinoline has been reported to date, with all structures being in non-Sohncke space groups and containing both atropisomers. The structure of the (1*P*)- and (1*M*)-enantiomers of biiq are presented in Figure 8.

Quaternized 1,1′-biisoquinolines are expected to have higher barriers to racemization (as discussed for 1,1′-biisoquinoline *N,N’*-dioxides in Section 3.1). Nevertheless, all quaternized and protonated 1,1′-biisoquinolines are in non-Sohncke space groups.

### 4.2. Chiroptical and Spectroscopic Properties 

The absorption spectrum of the parent compound **3** in EtOH exhibits maxima at 218, 274, 284, 312 and 324 nm with absorption coefficients of 77,900, 7370, 6920, 6480 and 9430 M^−1^ cm^−1^, respectively [41]. Various chiroptical correlations have been used to show that the (+)-forms of 1,1′-biisoquinolines possess the (1*M*) absolute configuration [42,45,46,49,50,51,114]

## 5. Complexes of 1,1′-Biisoquinolines

### 5.1. A Note on Stereochemical Nomenclature

In preparing this article, we became aware of the need to describe stereochemical arrangements in coordination entities containing multiple stereogenic features. Rigorous examination and consideration of the nomenclature challenges inherent in these situations has been limited, and the development and codification of required nomenclature has also lagged. As a consequence, ad hoc and mutually inconsistent approaches have been developed by individual research groups to address their particular needs [115,116,117,118,119,120,121,122,123]. It is clear that these situations are certainly not adequately covered in the standard recommendations from IUPAC [20,124]. This short section describes the approach we have adopted. 

**Figure 9:**
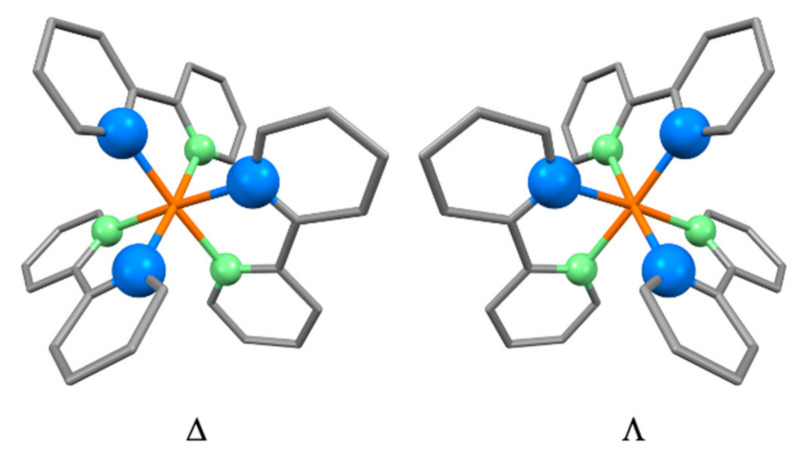
The absolute configurations of the two enantiomers of the [Ru(bpy)_3_]^2+^ cation. The green atoms of each bpy ligand are further away from the observer, and the blue atoms are closer. The cation resembles a propeller, with each blade being defined by the donor atoms of the bpy ligands. An anti-clockwise rotation of the right-hand structure will “dig” the propeller into the page. This is the Λ-isomer. The mirror-image propeller on the left must be rotated clockwise to do so, and is the denoted Δ-isomer.

Although IUPAC defines the descriptors Δ and Λ which describe the configuration of tris(bidentate) coordination entities, it neither tells you where to put the descriptor in the formula of the compound, nor the associated grammar (IR-9.3.4.1, IR-9.3.4.11, IR-9.3.4.12, IR-9.3.4.14) [124]. However, throughout the section dealing with stereochemical description of coordination entities (IR-9.3), the configuration index and other descriptors that relate to the configuration of ligands around the central atom are placed as prefixes to names and formulae. Due to this, and following general practice in the community, we prefer to place the configurational descriptors Δ and Λ before the name or formula of the coordination entity. For consistency with organic nomenclature [20], we have placed stereochemical descriptors preceding a name or formula in enclosing marks. Thus, the two enantiomers of the [Ru(bpy)_3_]^2+^ cation are denoted (Δ)-[Ru(bpy)_3_]^2+^ and (Λ)-[Ru(bpy)_3_]^2+^ (Figure 9).

A bulk material containing equal amounts of the two enantiomers of a compound is described as a racemate and can be denoted by the stereochemical descriptor *rac*- (P-91.2.1.1 [20]) combined with the stereodescriptor for the stereogenic site (P-93.1.3) [20]. The prefix *rac*- is defined as indicating the configuration of an entire molecule (P-93.1.3) [20]. This descriptor is particularly useful when applied to single crystals of materials in non-Sohncke space groups which contain equal amounts of the two enantiomers. However, while we could use the notation *rac*-(Δ)-[Ru(bpy)_3_]^2+^ as a description of a bulk material containing equal amounts of the Δ and Λ enantiomers, we prefer the notation (ΔΛ)-[Ru(bpy)_3_]^2+^ for such simple systems.

Where a stereogenic center (or axis, or plane) is associated with a ligand in a coordination entity, we have adopted the convention of associating the appropriate stereochemical descriptors using standard IUPAC protocols *with the ligand*. Although there are arguments for placing all of the stereochemical descriptors before the coordination entity, it will not always be clear which descriptor is associated with which ligand. Thus, we will use {(1*M*)-biiq} and {(1*P*)-biiq} to represent the chirality of biiq ligands in coordination entities. If the bulk material contains equal amounts of (1*M*)-biiq and (1*P*)-biiq we use the notation {(1*MP*)-biiq}. Remember that the term racemic refers to a property of a molecule or coordination entity as a whole: thus, the free ligand could be described as *rac*-(1*M*)-biiq but this notation should not be used within a formula for a related complex.

The question of relative stereochemistry is more complex and we enter *terra incognita* in the context of coordination entities. IUPAC has yet to examine and codify the ways of representing and distinguishing the subtly different stereochemical situations that are possible in these complex systems.

Consider the complex cation [Ru(biiq)_3_]^2+^, where this formulation encompasses any or all of the set of eight stereoisomers (four diastereoisomers and their enantiomers)

- (Δ)-[Ru{(1*M*)-biiq}_3_]^2+^ and (Λ)-[Ru{(1*P*)-biiq}_3_]^2+^
- (Δ)-[Ru{(1*M*)-biiq}_2_{(1*P*)-biiq}]^2+^ and (Λ)-[Ru{(1*M*)-biiq}{(1*P*)-biiq}_2_]^2+^
- (Δ)-[Ru{(1*M*)-biiq}{(1*P*)-biiq}_2_]^2+^ and (Λ)-[Ru{(1*M*)-biiq}_2_{(1*P*)-biiq}]^2+^
- (Λ)-[Ru{(1*M*)-biiq}_3_]^2+^ and (Δ)-[Ru{(1*P*)-biiq}_3_]^2+^

In a formula, {(1*MP*)-biiq} means that the biiq ligand is present in equal numbers in the bulk material with 1*M* or 1*P* chirality. This will not necessarily be the case in diastereoisomers and we need a notation for a ligand which may possess either chirality, but not necessarily with equal numbers with 1*M* or 1*P* chirality. This we denote in a formula as {(1*M/1P*)-biiq}. Similarly, ΔΛ denotes equal amounts of the Δ and Λ configurations, whereas Δ/Λ will be used for not necessarily equal amounts of either configuration, being present. 

The racemate of a particular diastereoisomer is represented by adding the *rac*- descriptor in front of a formula or name of a defined stereoisomer, so that *rac*-(Δ)-[Ru{(1*M*)-biiq}_3_]^2+^ refers to equal amounts of (Δ)-[Ru{(1*M*)-biiq}_3_]^2+^ and (Λ)-[Ru{(1*P*)-biiq}_3_]^2+^ being present. Note that *rac*- refers to the pairwise inversion of *all* of the stereogenic centers in a coordination entity. In principle, the racemate can also be defined using pairwise (or higher) groups of descriptors. Thus, (ΔΛ)-[Ru{(1*MP*)-biiq}_3_]^2+^ refers to the mixture of (Δ)-[Ru{(1*M*)-biiq}_3_]^2+^ and (Λ)-[Ru{(1*P*)-biiq}_3_]^2+^ being present. The order of symbols, for example in ΔΛ and *MP*, is significant in defining relative configurations of different stereogenic features (centers, axes, etc.) that are present in a particular diastereoisomer. (ΔΛ)-[Ru{(1*MP*)-biiq}_3_]^2+^ and (ΔΛ)-[Ru{(1*PM*)-biiq}_3_]^2+^, where the order of the ligand descriptors is reversed, refer to racemic mixtures of different diastereoisomers: *rac*-(Δ)-[Ru{(1*M*)-biiq}_3_]^2+^ and *rac*-(Δ)-[Ru{(1*P*)-biiq}_3_]^2+^, respectively. The potential for confusion in keeping track of the relative configurations that are present in a particular diastereoisomer, particularly when the descriptors are located in different parts of the formula or name, is a key reason for preferring the *rac*-descriptor over (ΔΛ) for systems where multiple diastereo-isomers are possible. Overall, these usages are best clarified with a few examples:

- (Δ)-[Ru{(1*M*)-biiq}_3_]^2+^ denotes a single enantiomer of a single diastereoisomer;
- *rac*-(Δ)-[Ru{(1*M*)-biiq}_3_]^2+^ denotes a racemic mixture of a single diastereoisomer (i.e., equal amounts of (Δ)-[Ru{(1*M*)-biiq}_3_]^2+^ and (Λ)-[Ru{(1*P*)-biiq}_3_]^2+^);
- *rel*-(Δ)-[Ru{(1*M*)-biiq}_3_]^2+^ denotes a mixture but not necessarily equal amounts of the two enantiomers of a single diastereoisomer: (Δ)-[Ru{(1*M*)-biiq}_3_]^2+^ and (Λ)-[Ru{(1*P*)-biiq}_3_]^2+^;
- (Δ/Λ)-[Ru{(1*M*)-biiq}_3_]^2+^ denotes variable amounts of the two diastereoisomers (Δ)-[Ru{(1*M*)-biiq}_3_]^2+^ and (Λ)-[Ru{(1*M*)-biiq}_3_]^2+^;
- (ΔΛ)-[Ru{(1*M*)-biiq}_3_]^2+^ denotes a 1:1 mixture of the diastereoisomers (Δ)-[Ru{(1*M*)-biiq}_3_]^2+^ and (Λ)-[Ru{(1*M*)-biiq}_3_]^2+^;
- (Δ)-[Ru{(1*MP*)-biiq}_3_]^2+^ denotes the mixture of diastereoisomers (Δ)-[Ru{(1*M*)-biiq}_3_]^2+^, (Δ)-[Ru{(1*M*)-biiq}_2_{(1*P*)-biiq}]^2+^, (Δ)-[Ru{(1*M*)-biiq}{(1*P*)-biiq}_2_]^2+^ and (Δ)-[Ru{(1*P*)-biiq}_3_]^2+^ in which the total numbers of (1*M*)-biiq and (1*P*)-biiq ligands in the bulk material are identical. This does not imply equal amounts of the different diastereoisomers;
- (Δ)-[Ru{(1*M/1P*)-biiq}_3_]^2+^ denotes a mixture of diastereoisomers (Δ)-[Ru{(1*M*)-biiq}_3_]^2+^, (Δ)-[Ru{(1*M*)-biiq}_2_{(1*P*)-biiq}]^2+^, (Δ)-[Ru{(1*M*)-biiq}{(1*P*)-biiq}_2_]^2+^, and (Δ)-[Ru{(1*P*)-biiq}_3_]^2+^ in which the total amount of (1*M*)-biiq and (1*P*)-biiq ligands is variable;
- (Δ/Λ)-[Ru{(1*MP*)-biiq}_3_]^2+^ denotes the mixture of eight stereoisomers listed earlier in which the total numbers of (1*M*)-biiq and (1*P*)-biiq ligands are identical but the numbers of Δ and Λ stereogenic centers may be different;
- (ΔΛ)-[Ru{(1*M/1P*)-biiq}_3_]^2+^ denotes the mixture of eight stereoisomers listed earlier in which the total numbers of (1*M*)-biiq and (1*P*)-biiq ligands are variable but the numbers of Δ and Λ stereogenic centers are identical
- (Δ/Λ)-[Ru{(1*M/1P*)-biiq}_3_]^2+^ denotes the mixture of eight diastereoisomers listed earlier in which the total numbers of (1*M*)-biiq and (1*P*)-biiq ligands and Δ and Λ stereogenic centers are freely variable.

The reaction of *rac*-(1M)-biiq with an achiral iron(II) salt will generate *rac*-(Δ)-[Fe{(1*M*)-biiq}_3_]^2+^. The notation *rac-*(Δ)-[Ru{(1*M*)-biiq}_2_{(1*P*)-biiq}]^2+^ can be used to describe a racemic mixture of the two enantiomers (Δ)-[Ru{(1*M*)-biiq}_2_{(1*P*)-biiq}]^2+^and (Λ)-[Ru{(1*M*)-biiq}{(1*P*)-biiq}_2_]^2+^. We also note that the use of the prefix *rel*- (P-93.1.4) [20] in *rel-*(Δ)-[Ru{(1*M*)-biiq}_2_{(1*P*)-biiq}]^2+^ is not equivalent as it does not imply equal amounts of the compounds.

This leads to descriptions such as (Δ)-[Ru{(1*P*)-biiq}(bpy)_2_]^2+^ for an enantiopure diastereoisomer and (ΔΛ)-[Ru{(1*P*)-biiq}(bpy)_2_]^2+^ for a 1:1 mixture of the diastereoisomers (Δ)-[Ru{(1*P*)-biiq}(bpy)_2_]^2+^ and (Λ)-[Ru{(1*P*)-biiq}(bpy)_2_]^2+^. Using the proposal for the use of *rac*- above, the enantiomeric pair (Δ)-[Ru{(1*P*)-biiq}(bpy)_2_]^2+^ and (Λ)-[Ru{(1*M*)-biiq}(bpy)_2_]^2+^ can be denoted *rac*-(Δ)-[Ru{(1*P*)-biiq}(bpy)_2_]^2+^. The formulae (ΔΛ)-[Ru{(1*MP*)-biiq}(bpy)_2_]^2+^ and (Δ/Λ)-[Ru{(1*M*/1*P*)-biiq}(bpy)_2_]^2+^ describes mixtures of multiple diastereoisomers and not a single enantiomeric pair. We contemplated extending this notation to the use of *ambo-* (P-93.1.4) [20] – but our heads exploded! 

The set of notations described above will be used where the stereochemical properties of the complexes are known or are of interest. Where no stereochemical information is known or implied, we simply use the abbreviation biiq.

### 5.2. Group 7 

**Figure 10:**
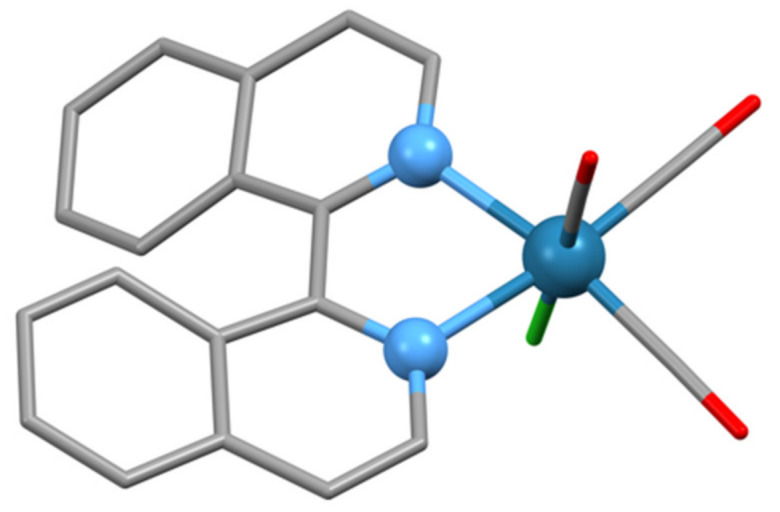
The structure of the [Re{(1*M*)-biiq)(CO)_3_Cl] molecule found in the lattice of [Re{(1*MP*)-biiq)(CO)_3_Cl]·PhMe. Hydrogen atoms have been omitted for clarity and the metal and nitrogen donor atoms are represented as balls.

The complex [Re{(1*MP*)-biiq)(CO)_3_Cl]·PhMe has been structurally characterized (Figure 10) and has the expected octahedral structure with two carbonyl ligands *trans* to the biiq *N*-donor atoms (CSD Refcode AYUPER, space group *P*$\bar{1}$) [125]. The lattice comprises equal numbers of complex molecules with (1*M*)-biiq and (1*P*)-biiq ligands with an N2–C1–C1′-N2′ torsion angle of −27.48°. Electrospray mass spectrometric (MS) studies of methanol solutions containing [Re(acac)_2_(Cl)(PPh_3_)] and biiq exhibited an ion assigned to the species {Re(biiq)(PPh_3_)}^+^ [126].

### 5.3. Group 8 

In the first paper describing the synthesis of biiq, Case reported that the compound did not give a typical ferroin coloration on treatment with iron(II) salts [81]. It was only many years later that iron complexes of biiq and its derivatives were prepared and characterized. The blue-green spin-crossover complexes [Fe(biiq)_3_]X_2_·2.5H_2_O (X = BF_4_, ClO_4_) are obtained directly from the reaction of biiq with the appropriate iron(II) salts [77]. Detailed magnetic and Mössbauer data for the compounds were reported, consistent with thermal population of the ^5^*T*_2_ state from the ^1^*A*_1_ ground state at higher temperatures. As might be expected for a sterically hindered heterocyclic diimine ligand, there is appreciable dissociation in acetone solutions of the complexes [77] and no complex ions were reported in electrospray MS studies of methanol solutions containing iron(II) chloride and biiq [126]. The complex [Fe_2_(H_2_O)_2_{6,6′,7,7′-(MeO)_4_biiq}_4_(μ-O)]Cl_4_·4H_2_O is reported to be an effective catalyst for the selective oxidation of primary and secondary alcohols to aldehydes and ketones, respectively [127].

**Figure 11:**
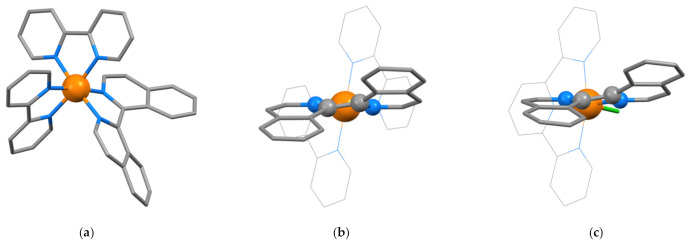
(**a**) The structure of the (Λ)-[Ru{(1*M*)-(biiq)}(bpy)_2_]^2+^ cation found in *rac*-(Δ)-[Ru{(1*P*)-(biiq)}(bpy)_2_](PF_6_)_2_ with hydrogen atoms omitted for clarity The lattice also contains the enantiomeric (Δ)-[Ru{(1*P*)-(biiq)}(bpy)_2_]^2+^ cation; (**b**) a projection of the (Λ)-[Ru{(1*M*)-(biiq)}(bpy)_2_]^2+^ cation in the N–Ru–N showing the positioning of C1 and C1′ above and below this plane. The bpy ligands are rendered as wireframe, the biiq as capped sticks and the five atoms defining the chirality of the chelate ring as balls, and all hydrogen atoms have been omitted for clarity; (**c**) the [Os{(1*M*)-biiq}Cl(tpy)]^+^ cation present in single crytsals obtained from rac-([Os{(1*MP*)-biiq}Cl(tpy)]Cl·H_2_O; the compound crystallized in homochiral crystals (all 1*M* or all 1*P*) in the space group *P*2_1_2_1_2_1_. The tpy ligand is rendered as wireframe, the biiq as capped sticks and the five atoms defining the chirality of the chelate ring as balls and all hydrogen atoms have been omitted for clarity.

The complex cation [Ru(biiq)(bpy)_2_]^2+^ was first described by Ashby et al. who also reported the solid-state structure of *rac*-(Δ)-[Ru{(1*P*)-biiq}(bpy)_2_](PF_6_)_2_ (CSD Refcode HAKPEO, HAKPEO10, space group *C*2/*c*, Figure 11a, N2–C1–C1′-N2′ torsion angles of 177.73, −24.12°) [128]. In the solid state, the two diastereoisomers (Δ)-[Ru{(1*P*)-biiq}(bpy)_2_](PF_6_)_2_ and (Λ)-[Ru{(1*M*)-biiq}(bpy)_2_](PF_6_)_2_ are present in the lattice (Ashby described the conformation of the chelate ring in terms of the λ and δ description for ethane-1,2-diamine chelate rings with λ corresponding to 1*M* and δ to 1*P*) (Figure 11b). Clemente has published a correction to the space group and a re-refinement of the structure reported (CSD Refcode HAKPEO11, space group *Ibca*, N2–C1–C1′-N2′ torsion angle of −24.14°) [129]. When the crystalline material is dissolved in CD_3_COCD_3_ at room temperature a 3:1 mixture of diastereoisomers ((Δ)-[Ru{(1*P*)-(biiq)}(bpy)_2_]^2+^ + (Λ)-[Ru{(1*M*)-(biiq)}(bpy)_2_]^2+^: (Λ)-[Ru{(1*P*)-(biiq)}(bpy)_2_]^2+^ + (Δ)-[Ru{(1*M*)-(biiq)}(bpy)_2_]^2+^) is observed in the ^1^H NMR spectrum, whereas dissolution at −80 °C gives a solution showing only the major pair of enantiomers. In solution, the related compound *rac*-(Δ)-[Ru{(1*P*)-biiq}(phen)_2_](PF_6_)_2_ is reported to exhibit two diastereoisomers in a 2:1 ratio [130]. The major and minor diastereoisomers of [Ru(biiq)(bpy)_2_]^2+^ interconvert at 80 °C. The original report by Ashby excluded Bailar and Rây-Dutt twisting mechanisms for the interconversion and it was suggested that one of the two biiq Ru–N bonds was broken, followed by either rotation about the C1–C1′ bond or a turnstile rotation, although isomerization via a planar biiq could not be excluded [128]. In a subsequent publication, the authors concluded that a monodentate biiq could not be involved, because the rate of racemization of [Ru(biiq)(bpy)_2_]Cl_2_ in D_2_O was essentially the same in the presence of 1M DCl and 1M LiCl and they proposed a transition state involving a bidentate planar biiq [72]. The authors extended the study to *rac*-(Δ)-[Os{(1*P*)-biiq}(bpy)_2_](PF_6_)_2_ which was crystallographically characterized (CSD Refcode YONHUE, space group *C*2*/c*, N2–C1–C1′-N2′ torsion angle −20.14°) and shown to exhibit the same favored diastereoisomers as the ruthenium analog in the solid state. Comparison of the rates of racemization for the ruthenium (ΔG^‡^_major––>minor_ 79 kJ mol^−1^ at 323 K) and osmium (ΔG^‡^_major––>minor_ 72 kJ mol^−1^ at 323 K) supported the proposal of the stabilization of a planar transition state as the strength of the M–N bond is expected to increase rather than decrease on descending the triad [131]. The space group of *rac*-(Δ)-[Os{(1*P*)-biiq}(bpy)_2_](PF_6_)_2_ has been revised by Clemente (CSD Refcode YONHUE01, space group *Ibca*, N2–C1–C1′-N2′ torsion angle −20.15°) [129]

**Scheme 3:**
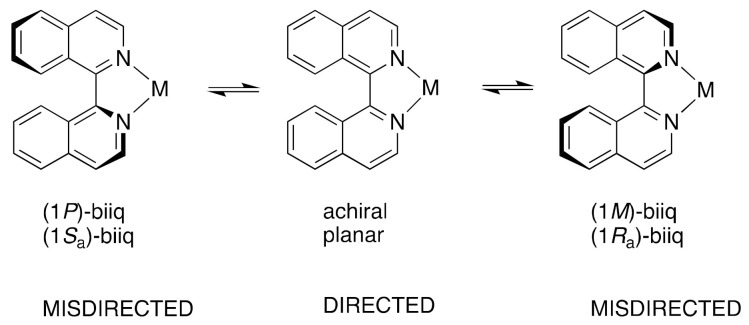
The mechanism proposed by Ashby for the racemization of coordinated 1,1′-biisoquinoline ligands. The term „misdirected” refers to the fact that the pair of vectors describing the orientation of the nitrogen lone pairs in the plane of the quinoline ring is not optimal for binding to the metal center. The various descriptions used for the chirality of the coordinated biiq ligands are also indicated.

The same phenomenon was also observed in the compounds [M{(1*MP*)-biiq}(η^6^-C_6_H_6_)X](PF_6_) (M = Ru, Os; X = Cl, I) and solid state structures were reported for [M{(1*MP*)-biiq}(η^6^-C_6_H_6_)Cl](PF_6_) (M = Ru, CSD Refcode QEWRAL, space group *P*$\bar{1}$, N2–C1–C1′-N2′ torsion angle −26.97°; M = Os, CSD Refcode QEWREP, space group *P*$\bar{1}$, N2–C1–C1′-N2′ torsion angle −26.47°) [132]. The racemization mechanism involving a planar biiq ligand was invoked (Scheme 3) and the independence from the ancillary ligands established by showing a similar mechanism for the compounds [M{(1*MP*)-biiq}(η^6^-C_6_H_6_)X](PF_6_) (M = Ru, Os; X = Cl, I) [132] and [M{(1*MP*)-biiq}Cl(tpy)](PF_6_) (M = Ru, Os; tpy = 2,2′:6′,2′’-terpyridine) [133]. The mechanism is of interest as an example of an inverse free-energy relationship in which the M–N bonds to the biiq ligand are stronger in the planar transition state than in the thermodynamically favored forms. Molecular mechanics parameters for ruthenium(II) oligopyridine and related compounds have been developed (MM3* force field in MacroModel combined with B3LYP frequency calculations) and the energy difference between the diastereoisomers (Δ)-[Ru{(1*P*)-(biiq)}(bpy)_2_]^2+^ and (Λ)-[Ru{(1*P*)-(biiq)}(bpy)_2_]^2+^ was calculated to be 3.0 kJ mol^−1^ in good accord with the experimental value of 2.7 kJ mol^−1^ [134]. Although the calculated energy of the planar transition state (100 kJ mol^−1^) implied in Scheme 3 was higher than the experimental value (70 kJ mol^−1^), the method confirmed that coordination of biiq to ruthenium lowers the isomerization barrier *for a mechanism involving a planar intermediate* [134]. The complexes [Ru(biiq)_2_Cl_2_] and [RuL_2_(L’)](ClO_4_)_2_ (L = bpy, phen, L’ = biiq; L = biiq, L’ = bpy, phen) and [Ru(biiq)_3_)](ClO_4_)_2_ have been prepared and their spectroscopic properties reported [135]. Solutions of salts of the complex cations [Ru(biiq)_3_)]^2+^, [Ru(biiq)_2_(bpy)]^2+^ and [Ru(biiq)(bpy)_2_]^2+^ in aqueous silver sol exhibit strong surface-enhanced resonance Raman scattering upon excitation in the MLCT bands. It is unfortunate that information was given neither on the diastereoisomers present nor on the anions, as these are expected to have a significant effect on this phenomenon [136]. Starke has reported some unusual collision-induced dissociation (CID) studies of homo- and heteroleptic ruthenium complexes with biiq ligands although from the formulations it is not clear exactly what complexes were studied [126].

**Scheme 4:**
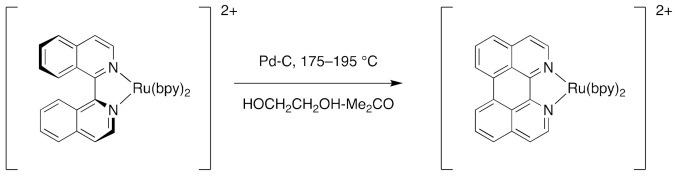
The steric interactions between H8 and H8′ in the coordinated biiq ligand in [Ru(biiq)(bpy)_2_](PF_6_)_2_ enables a facile dehydrogenation to generate a coordinated dap ligand in the complex [Ru(bpy)_2_(dap)](PF_6_)_2_.

The barrier to inversion of the coordinated biiq ligand relates primarily to the steric interaction between the H8 and H8′ atoms as well as the nitrogen lone pairs in the planar form. The conformation of the biiq ligand in its complexes can be seen as a balance between minimizing the H8–H8′ interactions (non-planar) and maximizing the M–N interactions (planar). Support for this comes from the observation that *rac*-[Ru(biiq)(bpy)_2_](PF_6_)_2_ is cleanly converted to [Ru(bpy)_2_(dap)](PF_6_)_2_ (dap = 1,12-diazaperylene) upon heating to 175–195 °C in HOCH_2_CH_2_OH-Me_2_CO in the presence of Pd-C (Scheme 4) [137].

The solid-state structures of [Os{(1*M*)-biiq}Cl(tpy)]Cl·H_2_O (CSD Refcode ACUDEH, Space group *P*2_1_2_1_2_1_, N2–C1–C1′–N2′ torsion angle −21.01°, Figure 11c) [133] and [Ru{(1*MP*)-biiq}Cl(tpy)](ClO_4_)·CH_3_CN (CSD Refcode YEZBEK, space group *P*2_1_/*c*, N2–C1–C1′–N2′ torsion angle 22.18°) [138] have been reported. The complex [Ru(biiq)(O)(Me_3_TACN)](ClO_4_) (Me_3_TACN = 1,4,7-trimethyl-1,4,7-triazacyclononane) is effective in the stoichiometric oxidation of alkenes to epoxides [139]. The compound [Ru(biiq)(phen)_2_](PF_6_)_2_ is very photostable and is only converted to [Ru(MeCN)_2_(phen)_2_](PF_6_)_2_ after several hours of irradiation with a 250 W halogen lamp [130].

A series of compounds including [Ru_2_(biiq)_2_(CO)_4_(μ-OAc)](OAc), [Ru(biiq)Cl(η^6^-*p*-cymene)]Cl and [Ru(biiq)Cl(η^6^-*p*-cymene)](BPh_4_) has been prepared and shown to be effective catalysts for the hydrogenation of alkenes [74]. The homoleptic compound [Ru(biiq)_3_](PF_6_)_2_ was also obtained in this work. The solid-state structure of [Ru{(1*MP*)-biiq)Cl(η^6^-*p*-cymene)](BPh_4_) (CSD Refcode GABGUM, space group *P*2_1_/*n*, N2–C1–C1′–N2′ torsion angle of −24.78°) [74] was also reported and the cation shown to be broadly similar to the [M(biiq)Cl(η^6^-C_6_H_6_)]^+^ (M = Ru, Os) cations previously described [132].

**Figure 12:**
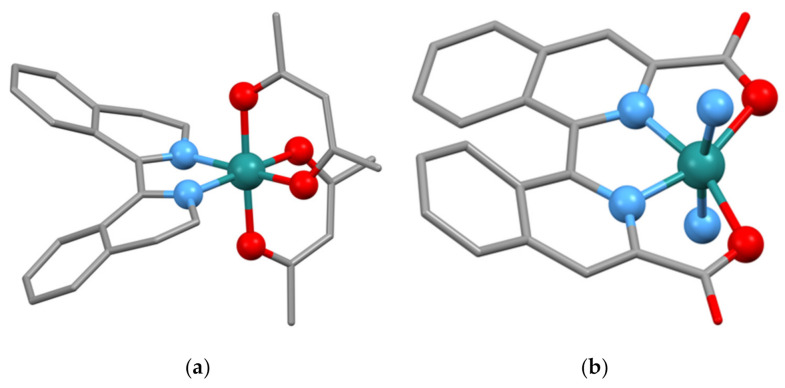
(**a**) The (Λ)-[Ru(acac)_2_{(1*P*)-biiq}]^+^ cation present in *rac*-(Δ)-[Ru(acac)_2_{(1*M*)-biiq}](ClO_4_). Equal numbers of (Δ)-[Ru(acac)_2_{(1*M*)-biiq}]^+^ cations are also present in the lattice. Hydrogen atoms omitted for clarity and donor atoms and the metal atoms are shown as balls; (**b**) The structure of the [Ru{(1*M*)-3,3′-(O_2_C-κ*O*)_2_biiq-κ^2^*N*}L_2_] molecule present in [Ru{(1*MP*)-3,3′-(O_2_C-κ*O*)_2_biiq-κ^2^*N*}L_2_]·2MeOH (L = 6-bromoisoquinoline). Equal numbers of [Ru{(1*P*)-3,3′-(O_2_C-κ*O*)_2_biiq-κ^2^*N*}L_2_] molecules are present in the lattice. Hydrogen atoms omitted for clarity and donor atoms and the metal atoms are shown as balls. Only the nitrogen donor atoms of the 6-bromoisoquinoline ligands are shown.

When the complexes [Ru(acac)_2_L](ClO_4_)*_n_* (L = 3,4-dihydro-1,1′-biisoquinoline or 3,3′,4,4′-tetrahydro-1,1′-biisoquinoline, *n* = 0 or 1) are treated with NEt_3_ under aerobic conditions, the ligands are fully aromatized and the compounds [Ru(acac)_2_(biiq)](ClO_4_)*_n_* (n = 0 or 1) are obtained [140]. The complex *rac*-(Δ)-[Ru(acac)_2_{(1*M*)-biiq}](ClO_4_) has been structurally characterized (CSD Refcode OGIVEI, space group *P*2/*n*, Figure 12a, N2–C1–C1′–N2′ torsion angle of 25.38°). The lattice contains equal numbers of the two diastereoisomeric cations (Δ)-[Ru(acac)_2_{(1*M*)-biiq)]^+^ and (Λ)-[Ru(acac)_2_{(1*P*)-biiq)]^+^ cations. The ruthenium(II) analogue *rac*-(Δ)-[Ru(acac)_2_{(1*P*)-biiq}] has also been structurally characterized (CSD Refcode OGIVUY, space group *P*2_1_/*c*, N2–C1–C1′–N2′ torsion angle of 18.51°). Interestingly, in this case the lattice contains equal numbers of the two diastereoisomeric cations (Λ)-[Ru(acac)_2_{(1*M*)-biiq)]^+^ and (Δ)-[Ru(acac)_2_{(1*P*)-biiq)]^+^ cations. It is unclear if the change in distereoisomeric selectivity in these two compounds is a case of diastereoisomerically selective crystallization for the two crystals determined, or if it reflects a more fundamental difference in thermodynamic stability of the complexes themselves. The Ru–O and Ru–N distances in the ruthenium(II) complex (Ru–O, 2.046–2.060 Å, Ru–N, 1.991, 1.993 Å) are significantly shorter than in the ruthenium(III) compound (Ru–O, 2.010, 2.011 Å, Ru–N, 2.037 Å) [140].

The complexes [Ru{3,3′-(O_2_C-κ*O*)_2_biiq-κ^2^*N*}L_2_] (L = 4-methylpyridine, 6-bromoisoquinoline) in which the {3,3′-(O_2_C)_2_biiq}^2−^ ligand acts as a planar tetradentate *N*,*N*’,*O*,*O*’-donor, have been prepared and shown to catalyze the oxidation of water in acidic media with Ce(IV) as the primary oxidant. Turnover numbers and frequencies are relatively modest, especially in comparison to related scaffolds based upon bpy or phen metal-binding domains [83]. The solid-state structure of [Ru{3,3′-(O_2_C)_2_biiq}L_2_]·2MeOH (L = 6-bromoisoquinoline) has been reported (CSD Refcode ABEVIP, space group *P*$\bar{1}$, Figure 12b) [83]. In the lattice there are equal numbers of complex molecules with (1*M*) and (1*P*) configurations of the biiq ligands, with an N2–C1–C1′–N2′ torsion angle of −15.35°. In contrast, the complex [Ru(biiq)Cl(tpy)](PF_6_)_2_ showed no activity in water oxidation studies [141]. 

### 5.4. Group 9 

No cobalt complexes of biiq appear to have been isolated although electrospray MS studies of methanol solutions containing CoCl_2_ and biiq observed an ion assigned to the species [Co(biiq)Cl]^+^ [126]

**Figure 13:**
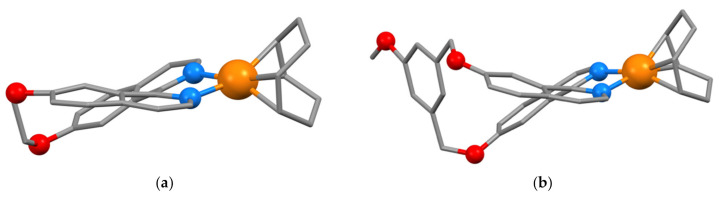
(**a**) The structure of the [Rh{(1*M*)-**7**}(cod)]^+^ cation found in [Rh{(1*MP*)-**7**}(cod)](ClO_4_)·Me_2_CO with hydrogen atoms omitted for clarity and non-carbon atoms as balls; (**b**) the structure of the [Rh{(1*M*)-**8**}(cod)]^+^ cation found in [Rh{(1*M*)-**8**}(cod)](ClO_4_)·Me_2_CO with hydrogen atoms omitted for clarity and non-carbon atoms as balls.

The reaction of [(cod)Rh(μ–Cl)_2_Rh(cod)] with 7,7′-(MeO)_2_biiq gives [Rh(cod){7,7′-(MeO)_2_biiq}](ClO_4_) [114]. This observation was the basis for a study of the coordination of the related enantiopure macrocyclic ligands (+)-**7** and (1*M*)- and (1*P*)-**8** with [(cod)Rh(μ–Cl)_2_Rh(cod)]. The reaction of (+)-**7** with [(cod)Rh(μ-Cl)_2_Rh(cod)] gave the structurally characterized complex [Rh{(1*MP*)-**7**}(cod)](ClO_4_)·Me_2_CO (CSD Refcode ZIJDIF, space group *P*2_1_/*a*, N2–C1–C1′-N2′ torsion angle of −28.49°, Figure 13a) in which the ligand has racemized and the lattice contains equal numbers of the [Rh{(1*M*)-**7**}(cod)]^+^ and [Rh{(1*P*)-**7**}(cod)]^+^ cations. In contrast, the configurationally stable ligands (1*M*)- and (1*P*)-**8** give homochiral complexes with retention of configuration. The compound [Rh{(1*M*)-**8**}(cod)](ClO_4_)·2CHCl_3_ (CSD Refcode ZIJDOL, space group *P*2_1_2_1_2_1_, N2–C1–C1′-N2′ torsion angle of −39.16°, Figure 13b) was structurally characterized, which also confirmed the 1*M* configuration for the (–) enantiomer of the ligand. [53]

**Figure 14:**
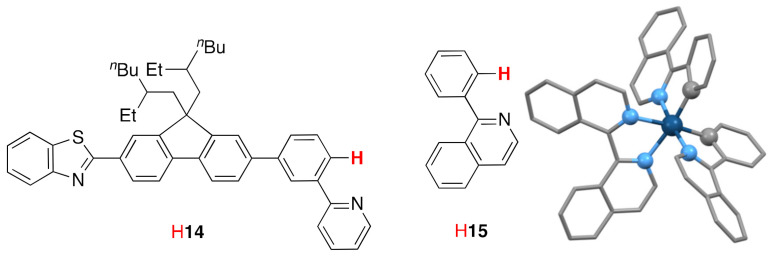
Two cyclometalating ligands (HC^N) which form strongly luminescent iridium(III) complexes of the type [Ir(biiq)(C^N)_2_](PF_6_) and the solid-state structure of the (Λ)-[Ir(**15**)_2_{(1*M*)-biiq}]^+^ cation present in *rac*-( Δ)[Ir(**15**)_2_{(1*M*)-biiq)](PF_6_). Hydrogen atoms have been omitted for clarity and donor atoms are represented as balls.

Cationic iridium(III) complexes with cyclometalating ligands are of interest for application as emitters in organic light-emitting diodes (OLEDs) and light-emitting electrochemical cells (LECs). The complex [Ir(**14**)_2_(biiq)](PF_6_) (Figure 14) has been prepared and the photophysical properties studied. The compound exhibits a strong orange-red emission at 672 nm [142]. The related complex [Ir(**15**)_2_(biiq)](PF_6_) (Figure 14) has been prepared and the photophysical properties as well as the solid-state structure reported (CSD Refcode REMQOQ, space group *P*$\bar{1}$, N2–C1–C1′–N2′ torsion angle −30.36°, Figure 14) [91]. In the solid-state structure of *rac*-(Δ)-[Ir(**15**)_2_{(1*M*)-biiq)](PF_6_), there are equal numbers of (Λ)-[Ir(**15**)_2_{(1*M*)-biiq}]^+^ and (Δ)-[Ir(**15**)_2_{(1*P*)-biiq}]^+^ cations.

### 5.5. Group 10 

**Figure 15:**
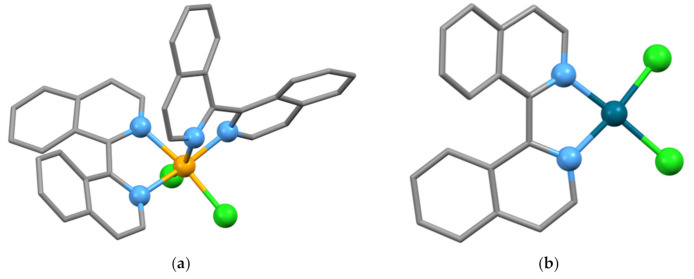
(**a**) A molecule of the complex *cis*-(Δ)-[Ni{{*M*)-biiq}{(*P*)-biiq}Cl_2_] present in *cis*-*rac*-(Δ)-[Ni{{1*M*)-biiq}{(1*P*)-biiq}Cl_2_]; equal numbers of *cis*-(Λ)-[Ni{{1*M*)-biiq}{(1*P*)-biiq}Cl_2_] molecules are present in the lattice. The donor atoms and nickel are shown as balls, and hydrogen atoms are omitted for clarity; (**b**) one of the two independent molecules of [Pd(biiq)Cl_2_] present in *cis*-*rac*-[Pd{(1*M*)-biiq}Cl_2_]. The molecule represented is *cis*-[Pd{(1*P*)-biiq}Cl_2_]. The other independent molecule possesses the *M* configuration. The donor atoms and palladium are shown as balls and hydrogen atoms omitted for clarity.

The bright green paramagnetic complex [Ni(biiq)_3_]^2+^ has been isolated from the reaction of biiq with [Ni(H_2_O)_6_](ClO_4_)_2_ in EtOH [77]. In contrast, the reaction with NiCl_2_·6H_2_O in *^n^*BuOH gives the structurally characterized compound [Ni(biiq)_2_Cl_2_] (CSD Refcode BAFRUY, space group *P*2_1_/*c*, N2–C1–C1′–N2′ torsion angles −40.11, 36.91°, Figure 15a) which has been shown to be an effective catalyst for the Suzuki coupling reaction [143]. Each molecule contains one biiq ligand with the 1*M* configuration and one with the 1*P* configuration leading to the full description *cis*-*rac*-(Δ)-[Ni{{1*M*)-biiq}{(1*P*)-biiq}Cl_2_] (remember that the metal is also a stereogenic center in an octahedral {M(N^N)_2 × 2_} species, where N^N is a chelating bidentate ligand). The complex [Pd(biiq)Cl_2_] has also been prepared and structurally characterized (CSD Refcode BAFSUZ, space group *P*2_1_/*c*, N2–C1–C1′–N2′ torsion angles −22.48, 27.64°, Figure 15b) and is effective as a catalyst in Suzuki and Heck reactions as well as in hydroxylation reactions [143]. The lattice of [Pd(biiq)Cl_2_] contains equal numbers of crystallographically independent [Pd[(1*M*)-biiq}Cl_2_] and [Pd{(1*P*)-biiq}Cl_2_] molecules.

**Scheme 5:**
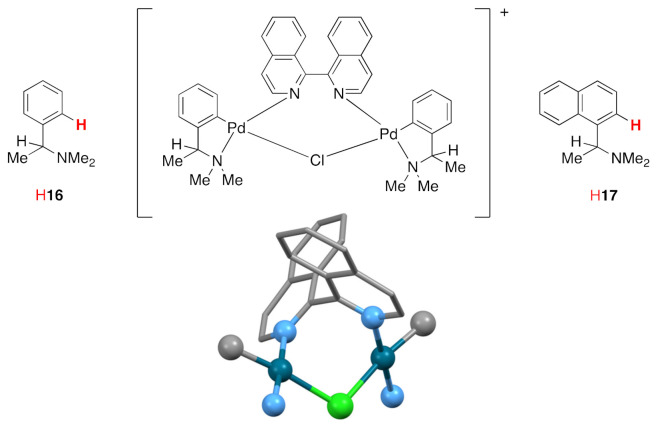
The chiral complexes [{(*R*)-**16**-κ*CN*}Pd(μ-Cl)_2_Pd{(*R*)-**16**-κ*CN*}] or [{(*S*)-**16**-κ*CN*}Pd(μ-Cl)_2_Pd{(*S*)-**16**-κ*CN*}] can be used to form the complexes [{(*R*)-**16**-κ*CN*}Pd(μ-Cl)(μ-(1*M*)-biiq-1κ*N*,2κ*N*’)Pd{(*R*)-**16**-κ*CN*}](ClO_4_) and [{(*S*)-**16**-κ*CN*}Pd(μ-Cl)(μ-(1*P*)-biiq-1κ*N*,2κ*N*’)Pd{(*S*)-**16**-κ*CN*}](ClO_4_) diastereoselectively. The solid-state structure of the [{(*S*)-**16**-κ*CN*}Pd(μ-Cl)(μ-(1*P*)-biiq-1κ*N*,2κ*N*’)Pd{(*S*)-**16**-κ*CN*}]^+^ cation in [{(*S*)-**16**-κ*CN*}Pd(μ-Cl)(μ-(1*P*)-biiq-1κ*N*,2κ*N*’)Pd{(*S*)-**16**-κ*CN*}](ClO_4_) is shown. Only the C and N donor atoms of the cyclometalated **16** ligands are shown. Hydrogen atoms have been omitted for clarity and donor atoms and the metal centers are depicted as balls.

The reaction of biiq with the chiral complexes [{(*R*)-**16**-κ*CN*}Pd(μ-Cl)_2_Pd{(*R*)-**16**-κ*CN*}] or [{(*S*)-**16**-κ*CN*}Pd(μ-Cl)_2_Pd{(*S*)-**16**-κ*CN*}] is diastereoisomer specific and gives the structurally characterized complexes [{(*R*)-**16**-κ*CN*}Pd(μ-Cl)(μ-(1*M*)-biiq-1κ*N*,2κ*N*’)Pd{(*R*)-**16**-κ*CN*}](ClO_4_) (CSD Refcode FOSVOY, space group *P*2_1_2_1_2_1_, N2–C1–C1′–N2′ torsion angles −93.72°) and [{(*S*)-**16**-κ*CN*}Pd(μ-Cl)(μ-(1*P*)-biiq-1κ*N*,2κ*N*’)Pd{(*S*)-**16**-κ*CN*}](ClO_4_) (CSD Refcode FOSVUE, space group *P*2_1_2_1_2_1_, N2–C1–C1′–N2′ torsion angle 91.59°) each containing a bridging biiq ligand (Scheme 5) [44]. The same strategy for the resolution of 8,8′-Me_2_biiq was less successful giving mixtures of diastereoisomers. However, using the corresponding reactions using H**17** exhibited a high diastereoselectivity and optically pure (1*P*)-8,8′-Me_2_biiq and (1*M*)-8,8′-Me_2_biiq were isolated from mother liquors or by reaction of [{(*R*)-**17**-κ*CN*}Pd(μ-Cl)(μ-(1*M*)-biiq-1κ*N*,2κ*N*’)Pd{(*R*)-**17**-κ*CN*}](ClO_4_) with dppe (1,2-bis(diphenylphosphano)ethane), respectively [43].

Electrospray and collision-induced dissociation MS studies of methanol solutions containing group 10 compounds and biiq have been reported [126]. The catalytic system [Pd(O_2_CCF_3_)_2_]–biiq has been shown experimentally to be moderately active in the reaction of 2,6-dimethoxybenzoic acid with MeCN to give 2,6-dimethoxyacetophenone; investigations at the DFT level were also reported [144].

The orange complex [Pt(biiq)Cl_2_] has been prepared by the direct reaction of K_2_[PtCl_4_] with biiq in water; the complex has been structurally characterized (CSD Refcode LEKXOO, space group *P*2_1_/*c*) and closely resembles the palladium analog (see above), with the lattice containing equal numbers of crystallographically independent [Pt[(1*M*)-biiq}Cl_2_] and [Pt{(1*P*)-biiq}Cl_2_] molecules with different torsion angles (35.18 and 41.12°) [145]. The reaction of [Pt(biiq)Cl_2_] with pyridine or 4-(*N*,*N*’-dimethylamino)pyridine (4-Me_2_Npy) gives the complexes [Pt(biiq-κ*N*)(py)_3_] and [Pt(biiq-κ*N*)(4-Me_2_Npy)_3_], respectively, each containing a monodentate biiq ligand. In the structurally characterized compound [Pt(biiq-κ*N*)(4-Me_2_Npy)_3_] (CSD Refcode LEKXUU, space group *P*$\bar{1}$) the torsion angle between the two isoquinoline ring systems of the biiq is 77.61° [145].

### 5.6. Group 11 

**Figure 16:**
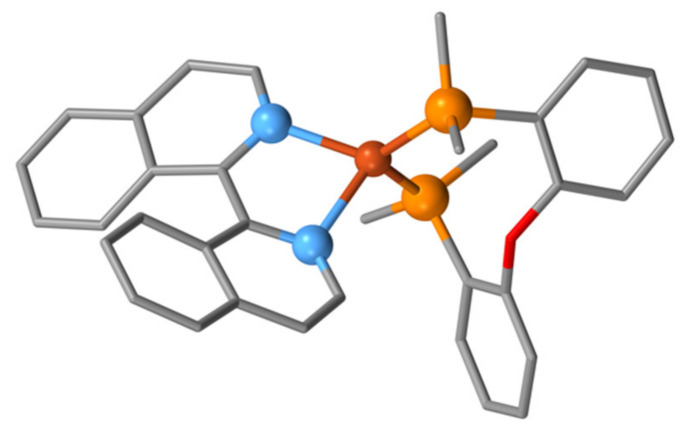
One of the enantiomeric [Cu(POP){(1*M*)-biiq}]^+^ cations present in [Cu(POP){(1*MP*)-biiq}](PF_6_) The donor atoms and copper are shown as balls and hydrogen atoms omitted for clarity with only the *ipso* carbon atoms of the Ph_2_P groups shown.

Until recently, the only biiq complex containing copper (outside the patent literature) was the red copper(I) compound [Cu(biiq)_2_](ClO_4_) obtained from the reaction of biiq with [Cu(CH_3_CN)_4_](ClO_4_) in MeCN [146]. The compound is relatively air-sensitive. We recently reported the preparation, characterization and photophysical properties of [Cu(POP)(biiq)](PF_6_) and [Cu(xantphos)(biiq)](PF_6_) (POP = bis(2-(diphenylphosphanyl)phenyl)ether, xantphos = (9,9-dimethyl-9H-xanthene-4,5-diyl)bis(diphenylphosphane) with a view to incorporating them as electroluminescent layers in light-emitting electrochemical cells. The NMR spectra indicate several concurrent dynamic processes involving inversion of both coordinated ligands. The solid-state structure of [Cu(POP){(1*MP*)-biiq}](PF_6_) has been determined (space group *P*$\bar{1}$, N2–C1–C1′–N2′ torsion angle −38.46) (Figure 16) [147]. 

Electrospray and collision-induced dissociation MS studies of methanol solutions containing CuCl_2_ and biiq exhibited ions assigned to the species [Cu(biiq)_2_Cl]^+^, [Cu(biiq)Cl]^+^ and [Cu_2_(biiq)_2_(Cl)_3_]^+^ [126]. In an extension to this work, heteroleptic and homoleptic complexes incorporating biiq and functionalized derivatives, bpy and phen were studied using ESMS and in situ complexation. Collision-induced decomposition measurements were used to determine the relatives tabilities of the copper(II) complexes [84]. 

**Figure 17:**
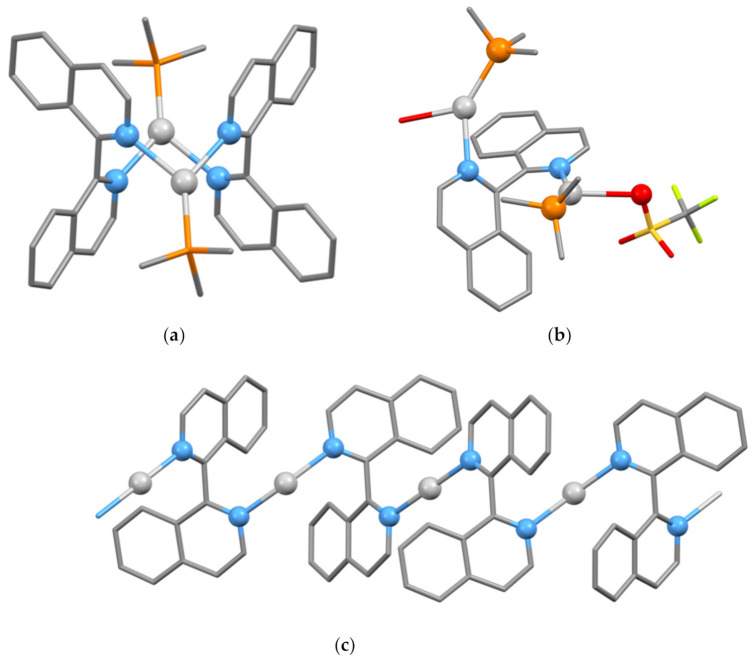
(**a**) The [(Ph_3_P)Ag(μ-biiq)_2_Ag(PPh_3_)]^2+^ cation present in [(Ph_3_P)Ag(μ-biiq)_2_Ag(PPh_3_)](ClO_4_)_2_·3Me_2_CO with donor atoms and silver atoms shown as balls, only the *ipso* carbon atoms of the Ph_3_P ligands are shown, and hydrogen atoms are omitted for clarity; (**b**) the compound [(CF_3_SO_3_)(Ph_3_P)Ag(μ-biiq)_2_Ag(OH_2_)(PPh_3_)]^+^ cation present in [(CF_3_SO_3_)(Ph_3_P)Ag(μ-biiq)_2_Ag(OH_2_)(PPh_3_)](CF_3_SO_3_)·CHCl_3_ with donor atoms and silver atoms shown as balls, only the *ipso* carbon atoms of the Ph_3_P ligands shown and hydrogen atoms omitted for clarity; (**c**) the one-dimensional [{Ag(μ-biiq)}*_n_*]*^n^*^+^ cation in [{Ag(μ-biiq)}*_n_*](CF_3_SO_3_)*_n_*·*n*H_2_O with donor atoms and silver atoms shown as balls and hydrogen atoms omitted for clarity.

As always, silver(I) complexes can be relied upon to provide new delights of structural diversity. Structurally characterized complexes containing silver(I) all contain two-, three- or four-coordinate silver(I) centers; no example of a chelating bidentate biiq ligand has been structurally characterized. Several structural motifs with biiq–Ag stoichiometries of 2:1, 1:2 and 1:1 have been reported. The compounds [{Ag(μ-biiq)_2_}*_n_*]X_2_ (X = BF_4_ or CF_3_SO_3_) of 2:1 stoichiometry are thought on the basis of conductivity data to be oligomeric or polymeric species containing silver centers coordinated to four biiq nitrogen donors [148]. The compounds of 2:2 stoichiometry [(Ph_3_P)Ag(μ-biiq-1κ*N*,2κ*N*’)_2_Ag(PPh_3_)](ClO_4_)_2_·3Me_2_CO (CSD Refcode YAQSIU, space group *P*$\bar{1}$, N2–C1–C1′-N2′, two crystallographically independent cations with torsion angles −100.28, 100.28° and 94.53, −94.53°, Figure 17a), [(MePh_2_P)Ag(μ-biiq-1κ*N*,2κ*N*’)_2_Ag(PMePh_2_)](ClO_4_)_2_·2CH_2_Cl_2_ (CSD Refcode YAQSOA, space group *P*$\bar{1}$, N2–C1–C1′–N2′ torsion angle −79.73, 79.73°) and [(MePh_2_P)Ag(μ-biiq-1κ*N*,2κ*N*’)_2_Ag(PMePh_2_)](CF_3_SO_3_)_2_·2CH_2_Cl_2_ (CSD Refcode YAQSUG, space group *P*2_1_*/c*, N2–C1–C1′–N2′ torsion angles −98.03, 98.03°) all contain discrete, centrosymmetric dinuclear cations with two three-coordinate silver centers [148]. A similar dinuclear {Ag(μ-biiq-1κ*N*,2κ*N*’)_2_Ag} core is present in [Ag_2_(μ-biiq-1κ*N*,2κ*N*’)_2_](CF_3_SO_3_)_2_·4Me_2_CO which exhibits many short Ag…O contacts (CSD Refcode YAQRUF, space group *C*2*/c*, N2–C1–C1′–N2′ torsion angles 67.84, 66.00°) and [(CF_3_SO_3_-κ*O*)Ag(μ-biiq)_2_Ag(CF_3_SO_3_-κ*O*)]·2Me_2_CO (CSD Refcode YAQSAM, space group *Pbcn*, N2–C1–C1′–N2′ torsion angles 68.22, 79.95°) [148]. In [Ag_2_{(μ-biiq-1κ*N*,2κ*N*’)_2_](CF_3_SO_3_)_2_·4Me_2_CO and [(CF_3_SO_3_-κ*O*)Ag(μ-biiq)_2_Ag(CF_3_SO_3_-κ*O*)]·2Me_2_CO, equal numbers of the homochiral cations [Ag_2_{(1*P*)-μ-biiq-1κ*N*,2κ*N*’}_2_]^2+^ and [Ag_2_{(1*M*)-μ-biiq-1κ*N*,2κ*N*’}_2_]^2+^ are present, whereas [(Ph_3_P)Ag(μ-biiq-1κ*N*,2κ*N*’)_2_Ag(PPh_3_)](ClO_4_)_2_·3Me_2_CO, [(MePh_2_P)Ag(μ-biiq-1κ*N*,2κ*N*’)_2_Ag(PMePh_2_)](ClO_4_)_2_·2CH_2_Cl_2_ and [(MePh_2_P)Ag(μ-biiq-1κ*N*,2κ*N*’)_2_Ag(PMePh_2_)](CF_3_SO_3_)_2_·2CH_2_Cl_2_ contain heterochiral [Ag_2_{(1*M*)-μ-biiq-1κ*N*,2κ*N*’}{(1*P*)-μ-biiq-1κ*N*,2κ*N*’}]^2+^ cations. 

A 1:2 stoichiometry is found in the compound [(CF_3_SO_3_)(Ph_3_P)Ag(μ-biiq-1κ*N*,2κ*N*’)_2_Ag(OH_2_)(PPh_3_)](CF_3_SO_3_)·CHCl_3_ (CSD Refcode YAQROZ, space group *P*$\bar{1}$, N2–C1–C1′–N2′ torsion angle 113.05°, Figure 17b) and related species with other phosphane ligands and anions [148]. The complexes [{Ag(μ-biiq)}*_n_*]X (X = BF_4_ or CF_3_SO_3_)·H_2_O are one-dimensional polymers; the solid-state structure of [{Ag(μ-biiq-1κ*N*,2κ*N*’)}*_n_*](CF_3_SO_3_)*_n_*·*n*H_2_O (CSD Refcode YAQSEQ, space group *C*2/*c*, N2–C1–C1′–N2′ torsion angle 111.76°, Figure 17c) has been reported [148].

Electrospray MS studies of methanol solutions containing Ag(BF_4_) and biiq exhibited ions assigned to the species [Ag(biiq)_2_]^+^ and [Ag(biiq)]^+^; if bpy (**1**) or phen (**2**) were also in the solution the ions [Ag(biiq)(**1**)]^+^ and [Ag(biiq)(**2**)]^+^ were observed [126]. Subsequent studies together with DFT level calculations were reported for substituted biiq ligands [85].

**Figure 18:**
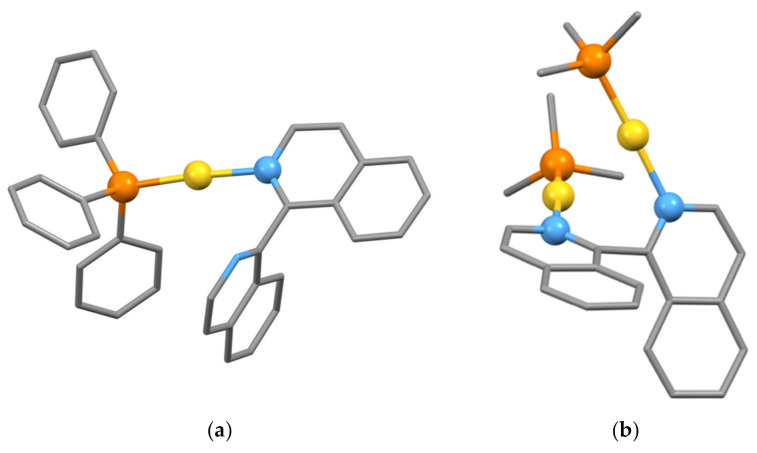
(**a**) In the compound [Au(biiq-κ*N*)(PPh_3_)](CF_3_SO_3_) the biiq ligand is monodentate and coordinates to a single near-linear (∠P–Au–N, 175.84°), two coordinate gold center with the donor atoms and the gold atoms represented as balls and hydrogen atoms omitted for clarity; (**b**) whereas a bridging mode is found in [(Me_3_P)Au(biiq-1κ*N*,2κ*N*’)Au(PMe_3_)](CF_3_SO_3_)_2_ with the donor atoms and the gold atoms represented as balls and hydrogen atoms omitted for clarity.

The preferred geometry for gold(I) is linear two-coordinate and the biiq ligand cannot, therefore, coordinate in a chelating bidentate mode. Two types of discrete complexes are found with gold(I) centers. A monodentate biiq ligand is found in [Au(biiq-κ*N*)(C_6_F_5_)] and also in the structurally characterized compound [Au(biiq-κ*N*)(PPh_3_)](CF_3_SO_3_) (CSD Refcode DEGMUZ, space group *P*$\bar{1}$, ∠P–Au–N, 175.84°, N2–C1–C1′–N2′ torsion angle −100.32°, Figure 18a) [149]. In contrast, bridging biiq ligands are found in the compounds [(Me_3_P)Au(biiq-1κ*N*,2κ*N*’)Au(PMe_3_)](CF_3_SO_3_)_2_ (CSD Refcode DEGMIN, space group *Pca*2_1_, ∠P–Au–N, 175.29, 176.24°, N2–C1–C1′–N2′ torsion angle 65.17°, Figure 18b), [(MePh_2_P)Au(biiq-1κ*N*,2κ*N*^’^)Au(PMePh_2_)](CF_3_SO_3_)_2_ (CSD Refcode DEGMOT, space group *P*$\bar{1}$, ∠P–Au–N, 175.63, 176.89°, N2–C1–C1′–N2′ torsion angle 95.67°) and [(C_6_F_5_)Au(biiq-1κ*N*,2κ*N*^’^)Au(C_6_F_5_)] (CSD Refcode DEGNAG, space group *C*2*/c*, ∠C–Au–N 178.96°, N2–C1–C1′–N2′ torsion angle 80.10°) [149].

### 5.7. Group 12 

Electrospray MS studies of methanol solutions containing Zn(OAc)_2_ and biiq exhibited ions assigned to the species [Zn(biiq)_2_(OAc)]^+^ and [Zn(biiq)(OAc)]^+^. In contrast, similar studies using Cd(NO_3_)_2_ gave species assigned to [Cd(biiq)(OCH_3_)_2_]^+^. The formula reported for [Zn(biiq)(OAc)]^+^ and the masses for [Zn(biiq)(OAc)]^+^ and [Cd(biiq)(OCH_3_)_2_]^+^ are incorrect and these formulations must be regarded as suspect [126].

## 6. Conclusions

This review has attempted to present a comprehensive discussion of the chemistry, particularly the coordination chemistry of 1,1′-biisoquinoline. The consequences of coordination of these atropisomeric ligands identified the need for a refinement and clarification of the stereochemical nomenclature for complexes with multiple stereogenic sites and we offer a first approach.

## Figures and Tables

**Table 1:** Structurally characterized 1,1′-biisoquinolines.

Compound	Refcode	N2–C1–C1′-N2′/°	Space Group	Reference
*rac*-(1*M*)-biiq	VEMWOC	−90.96	*I*4_1_/a	[108]
*rac*-(1*M*)-8,8′-Me_2_biiq	GORTUC	−101.18	$R\bar{3}$	[46]
*rac*-(1*M*)-8,8′-Me_2_biiq	GORTUC01	−101.18	*R* $\bar{3}$	[45]
*rac*-(1*M*)-8,8′-Et_2_biiq	GORVAK	100.63	*Pbca*	[46]
*rac*-(1*M*)-8,8′-Et_2_biiq	GORVAK01	100.63	*Pbca*	[45]
*rac*-(1*M*)-4,4′-(EtO)_2_biiq	MUKDEC	−119.01	*C*2/*c*	[87]
*rac*-(1*M*)-3,3,4,4′-(4-MeOC_6_H_4_)_2_biiq	RUKLUH	−124.67	*P* $\bar{1}$	[66]
*rac*-(1*M*)-**9**	MOCWIL	63.79	*P*2_1_/*n*	[54]

**Table 2:** Structurally characterized quaternized 1,1′-biisoquinolines and related species.

Compound	Refcode	N2–C1–C1′-N2′/°	Space Group	Reference
*rac-*(1*M*)-[H_2_(4,4′-(HO)_2_biiq)](Cl)_2_	MUKDAY	46.88	*C*2/*c*	[87]
*rac*-(1*M*)-(**10**)(I)_2_·H_2_O	LALXUR	−107.81	*A*2/*a*	[110]
*rac*-(1*M*)-(**11**)(Br)_2_	KOTCAX	−37.23	*I*4_1_/*a*	[110]
*rac*-(1*M*)-(**12**)(Br)_2_·H_2_O	KOTCEB	−59.74	*P* $\bar{1}$	[110]
(1*P*)-biiq *N*,*N*’-dioxide	HIMGUF	101.64	*P*2_1_	[98]
